# Diffuse alveolar hemorrhage following inhaled sevoflurane: A rare complication of inhalational anesthesia

**DOI:** 10.5578/tt.20239611

**Published:** 2023-12-07

**Authors:** Burcu Baran Ketencioğlu, Meliha Hastekkeşin, Nur Aleyna YETKİN, Nuri TUTAR

**Affiliations:** 1 Department of Chest Diseases, Erciyes University Faculty of Medicine, Kayseri, Türkiye

## Abstract

**ABSTRACT**

**
Latest status of non-tuberculous mycobacteria prevalence in Türkiye and the world: Systematic review
**

Non-tuberculous mycobacteria (NTM) can cause diseases not only in indivi- duals with compromised immune systems but also in those with normal immune function. This study aimed to compare the prevalence of NTM in Türkiye and worldwide between 2012 and 2022. This study was designed following the guidelines outlined in the Preferred Reporting Items for Systematic Reviews and Meta-Analyses (PRISMA) procedure. A systematic search was conducted between January 2012 and September 2022 using different electronic databases, including Pubmed, Medline, Embase, Web of Science, Ebsco, Scopus, Türk Medline, and Google Scholar. During the litera- ture review process, titles and abstracts were examined and the full texts of the studies were accessed. In 13 research articles from Türkiye included in the study, a total of 17.293 samples were studied and a total of 1304 NTM (7.54%) strains were isolated from these samples. Among the 1304 NTM strains reported from Türkiye, the top three most frequently isolated species were M. abscessus (29.83%), M. lentiflavum (14.97%), M. fortuitum (14.38%). In 35 studies included from around the world, a total of 512.626 samples were studied and a total of 12.631 NTM (2.46%) strains were isola- ted from these samples. Among the 12631 NTM strains isolated, the top three most frequently isolated species were M. intracellulare (28.13%), M. avium (17.70%) and M. abscessus (14.88%). This study unveiled the global preva- lence of NTM-infected patients, detailing species distribution and microbiolo- gical diagnostic methods. Variations in NTM spread were observed, influen- ced by diverse factors.

**Key words:** Non-tuberculous mycobacteria; prevalence; Mycobacterium abs- cessus; Mycobacterium avium

**ÖZ**

**
Türkiye ve dünyada tüberküloz dışı mikobakteri prevalansında son durum: Sistematik derleme
**

Tüberküloz dışı mikobakteriler (TDM), özellikle immün sistemi baskılanmış bireylerde hastalıklara sebep olmakla beraber, bağışıklık sistemi normal kişiler- de de hastalık oluşturmaktadır. Bu çalışmanın amacı 2012-2022 yılları arasın-

da Türkiye ve dünyadaki TDM prevalansının karşılaştırılmasıdır. Bu çalışma, Sistematik Derlemeler ve Meta Analizler için Tercih Edilen Raporlama Ögeleri (PRISMA) prosedürü kuralları baz alınarak planlanmıştır. Ocak 2012-Eylül 2022 tarihleri arasında Pubmed, Medline, Embase, Web of Science, Ebsco, Scopus, Türk Medline ve Google Scholar dahil olmak üzere farklı elektronik veri tabanları kullanarak sistematik bir tarama gerçekleştirilmiştir. Literatür tarama sürecinde başlık ve özetler incelenmiş ve çalışmaların tam metin- lerine ulaşılmıştır. Türkiye’den çalışmaya dahil edilen 13 araştırma makalesinde toplam 17,293 örnek ile çalışılmış ve bu örneklerden toplam 1304 TDM (%7,54) suşu izole edilmiştir. Türkiye’den bildirilen 1304 TDM suşu içinde en sık izole edilen ilk üç tür sırasıyla;

M. abscessus (%29,83), M. lentiflavum (%14,97), M. fortuitum (%14,38) olarak saptanmıştır. Dünya genelinden dahil edilen 35 çalışmada toplam 512,626 örnekle çalışılmış ve bu örneklerden toplam 12631 TDM (%2,46) suşu izole edilmiştir. İzole edilen 12,631 TDM suşu içinde en sık izole edilen ilk üç türün sırasıyla; M. intracellulare (%28,13), M. avium (%17,70), M. abscessus (%14,88) olduğu saptanmıştır. Bu çalışma sonucunda, dünya çapında TDM’ler ile enfekte hastaların prevalansı, türlerine göre dağılım ve mik- robiyolojik tanı yöntemleri gözler önüne sermiş olup, TDM’lerin yayılımının pek çok farklı faktöre bağlı olarak değiştiği görülmüştür.

**Anahtar kelimeler:** Tüberküloz dışı mikobakteriler; prevalans; mycobacterium abscessus; mycobacterium avium

**ABSTRACT**

**
Diffuse alveolar hemorrhage following inhaled sevoflurane: A rare complication of inhalational anesthesia
**

*
Sevoflurane is a commonly used inhalational anesthetic agent for inducing and maintaining general anesthesia. However, it has been associated with a rare but serious pulmonary condition known as diffuse alveolar hemorrhage (DAH). DAH is characterized by decreased hemoglobin levels, diffuse pul- monary infiltration, and respiratory failure with hypoxemia. We present a case of DAH in a healthy young adult who experienced this condition following general anesthesia with inhaled sevoflurane during an uncomplicated ortho- pedic procedure. Notably, there were no other risk factors or known causes that could account for the development of DAH in this patient.
*

**Key words:** *Diffuse alveolar hemorrhage; hemoptysis; inhaled anesthetics; inhaled sevoflurane
*

**ÖZ**

**
inhale sevofluran sonrası diffüz alveolar hemoraji: inhalasyon anestezisinin nadir bir komplikasyonu
**

*
Sevofluran, genel anesteziyi indüklemek ve sürdürmek için yaygın olarak kullanılan bir inhalasyon anestezik ajandır. Bununla birlikte, diffüz alveolar hemoraji (DAH) olarak bilinen nadir fakat ciddi bir pulmoner durumla ilişki- lendirilmiştir. DAH, azalmış hemoglobin seviyeleri, yaygın pulmoner infiltras- yon ve hipoksemi ile solunum yetmezliği ile karakterizedir. Komplike olmayan bir ortopedik prosedür sırasında inhale sevofluran ile genel anestezi sonrası DAH gelişen sağlıklı genç erişkin vakasını sunuyoruz. Özellikle, bu hastada DAH gelişimini açıklayabilecek başka risk faktörü veya bilinen neden mevcut değildir.
*

**Anahtar kelimeler:** *Diffüz alveolar hemoraji; hemoptizi; inhale anestezikler; inhale sevofluran
*

## INTRODUCTION

Anesthetic gases (nitrous oxide, halothane, isoflu- rane, desflurane, and sevoflurane), also known as inhaled anesthetics, are regularly used in the clinical setting because of their chemical properties that allow an agent to enter the arterial blood rapidly through the pulmonary circulation, compared to the more circuitous route of venous circulation (1). Diffuse alveolar hemorrhage (DAH) is a life-threaten- ing pulmonary pathology that includes decreased hemoglobin, diffuse pulmonary infiltration, and hypoxemic respiratory failure (2). Common causes of DAH are vasculitis, infections, anticoagulant use, and drugs (3). Drugs known to cause DAH include ami- odarone, amphetamine, cytotoxic agents, nitrofuran- toin, and sirolimus and case reports implicate the inhaled anesthetic sevoflurane (4-9). In this article, a case of DAH in a healthy young adult after general anesthesia with inhaled sevoflurane during an uncomplicated orthopedic procedure in the absence of other risk factors or known causes is presented.

## CASE REPORT

The patient is a 23-year-old male medical student with no known chronic disease. There was a three pack/year smoking history. After a sports injury, the patient planned to have an arthroscopic slap repair

on the right shoulder. The preoperative COVID PCR test was negative, respiratory system examination

was normal, and SpO2 measured 98% in arterial blood gas taken in room air. Inhaled sevoflurane and

intravenous propofol and fentanyl were used as anesthetics during surgery. The empirical antibiotic cefazolin was given in the perioperative period.

After the operation, the patient was extubated and taken to a recovery room without any problems. However, the patient developed hypoxemia and hemoptysis approximately one hour after he was taken to the room. The patient had no fever, the saturation measured from the finger decreased to 75% in room air, pulse rate was 112/min, respiratory rate was 28/min, and blood pressure was 120/70 mmHg. In the respiratory system examination, rales were heard bilaterally, especially in the lung bases. In the hemogram, there were leukocytosis (15.71 x 10^9^/L), a decrease of about three units in the hemoglobin value, which was in normal preoperative values (from 16.1 to 13.5 g/dL), and normal platelet count (185 x 10^9^/L). Bleeding parameters and kidney and liver functions were within the normal range. The C-reactive protein value was increased (64.2 mg/L), but the procalcitonin value was within the normal range (Table 1). A bilateral patchy increase in density was observed in the chest X-ray (Figure 1).

**Table:** 

**Table 1.** Pre-operative and post-operative laboratory evaluation of the patient			
**Laboratory tests**	**Normal range**	**Pre-operative values**	**Post-operative values**
Leukocyte count	4.8-10.7 103/µL	7.52	10.47
Hemoglobin	14-18 g/dL	**16.1**	**13.9**
Platelet count	130-400 103/µL	247	190
Neutrophil count	2.2-4.8 103/µL	3.62	6.57
Monocyte count	0.3-0.8 103/µL	0.59	0.51
Eosinophil count	0-0.2 103/µL	0.14	0.25
Basophil count	0-0.1 103/µL	0.05	0.03
Prothrombin time	10-14 sec	11.6	11.4
INR	0.8-1.2	0.97	0.96
Blood urea nitrogen	6-20 mg/dL	7	8
Creatinine	0.5-1.20 mg/dL	0.95	0.8
ALT	0-40 u/L	22	14
AST	0-41 u/L	15	17
Albumin	3.5-5.2 g/dL	5.08	4.04
C-reactive protein	0-5 mg/L	1.68	64.12
Procalcitonin	0-0.5 ng/mL	-	0.43
ALT: Alanine aminotransferase, AST: Aspartate aminotransferase, INR: International normalized ratio.

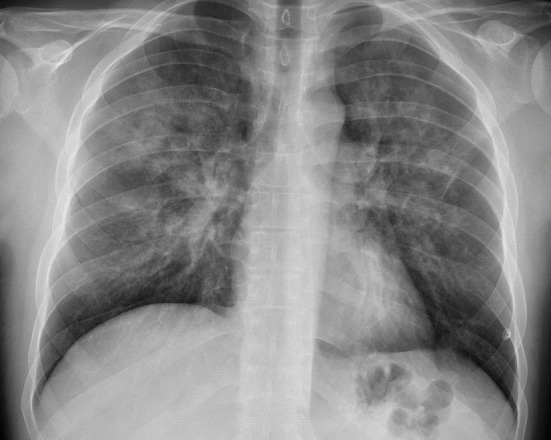

**Figure 1.** Chest X-ray at the time of onset of symptoms in the first hour postoperatively, shows increased bilateral cen- tral-dominated densities in the upper and middle zones.

In contrast-enhanced thorax computed tomography (CT) imaging, bilateral peribronchovascular patchy ground-glass areas were observed when the pulmonary artery and its branches were open (Figure 2). The patient was re-tested for COVID-19 along with the respiratory viral panel and the test results were negative. The patient’s saturation was

>90% with a nasal cannula and 4 L/min oxygen support. When flexible video bronchoscopy was

performed on the patient, there was no focus of active bleeding, but the mucosa was erythematous. Microbiological agents did not grow in the bronchoalveolar lavage material taken during bronchoscopy, and macrophages loaded with hemosiderin were observed in cytology. The patient was started on 100 mg/day intravenous methylprednisolone treatment with a preliminary diagnosis of DAH in the early period. Empirical antibiotic therapy was continued. Serological tests were negative for HIV, viral hepatitis, vasculitis, and connective tissue disease (anti-neutrophil cytoplasmic antibodies, proteinase 3, myeloperoxidase, rheumatoid factor, antinuclear antibodies, anti-dsDNA, anti-glomerular basement membrane antibody, and cryoglobulins). Complement levels (C3, C4) were within normal limits. Urinalysis was negative for red blood cells and protein. The dose of methylprednisolone was tapered and discontinued after three days of 100 mg/ day administration. On the fourth day, regression was observed in the control PA chest X-ray and the patient was discharged from the hospital. In the control thorax CT taken one month later, the lesions had completely healed (Figure 3). During the two- year follow-up of the patient, his complaints did not recur, and he stopped smoking during this period.

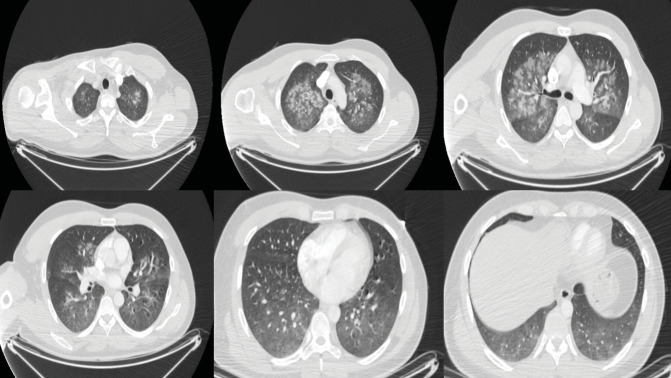

**Figure 2.** Axiel CT images show bilateral diffuse alveolar opacities, with a more central location in the upper lobes and a more diffuse appearance in the lower lobes.

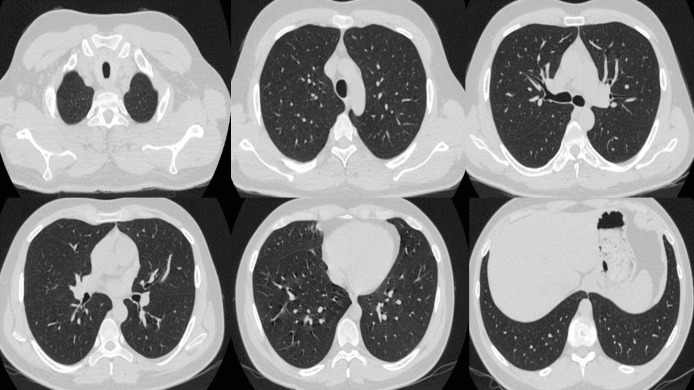

**Figure 3.** In the axial CT images obtained during the follow-up one month after discharge, it is evident that the pulmonary lesions have undergone complete resolution.

## DISCUSSION

Sevoflurane is an inhalational anesthetic agent widely used for the induction and maintenance of general anesthesia. The drug has a fast wash-out time compared to other agents in its class due to its lower blood solubility and blood-gas partition coefficient (10). Common side effects are nausea (25%), vomiting (18%), hypotension (4-11%), and bradyarrhythmia (3-5%). Respiratory system side effects include cough (5-11%), apnea (2-6%), laryngeal spasm (2-8%), and respiratory depression. Airway thermal injury and acute respiratory distress syndrome/acute lung injury have been reported (11,12) and sevoflurane-induced DAH cases have been published in recent years (4-9).

When the literature was evaluated, postoperative DAH was described in patients receiving inhaled sevoflurane. The first of these, a patient with end- stage renal disease and a history of cocaine use, developed DAH immediately after elective cataract surgery in which he received sevoflurane, propofol, and midazolam as anesthetics (4). The authors considered the patient’s kidney disease and coagulopathy and platelet dysfunction secondary to cocaine use as triggers for this condition. In a second case report, a 25-year-old male patient with

obstructive sleep apnea developed DAH immediately after tonsillectomy and received desflurane gas as an anesthetic (13). The authors suggested that in this case, the patient’s severe snoring may have damaged the alveolar wall causing barotrauma during general anesthesia, which may have caused DAH. In another case, DAH developed immediately after perirectal pilonidal cyst surgery at the age of 31. Although this patient did not have a chronic disease, he used cannabis, and urine toxicology screening was positive (5). Recently, a 21-year-old healthy male patient, similar to our case, developed postoperative DAH secondary to sevoflurane inhalation during an uncomplicated orthopedic procedure in the absence of any accompanying risk factors or other causes (9). In all cases, the clinical improvement of the patients with symptomatic treatment and no recurrence was observed. Our patient developed DAH without clinical or serological evidence of an underlying systemic disease. In his history, there was no positive finding other than smoking, and infection, vasculitis, malignancy, and other causes were excluded. Considering its acute onset in the postoperative period and limited drug exposure, inhaled sevoflurane was considered to be the most likely causative agent for DAH. Alveolar hemorrhage secondary to inhaled

sevoflurane should be kept in mind in cases of postoperative acute respiratory distress and pulmonary hemorrhage in which other etiologies have been excluded.

## CONFLICT of INTEREST

The authors have no conflict of interest to declare.

## AUTHORSHIP CONTRIBUTIONS

Concept/Design: BBK, MH Analysis/Interpretation: MMK, MH, NAY Data acqusition: BBK, NT

Writing: MH, NAY

Clinical Revision: BBK, NAY Final Approval: BBK, MH

## REFERENCES

1. *
  Clar DT, Patel S, Richards JR. Anesthetic gases. (2022) In: StatPearls [Internet]. Treasure Island (FL). StatPearls Publishing; 2023 Jan-.
  *
2. *
  Hao D, Basnet S, Melnick S, Kim J. Negative pressure pul- monary edema-related diffuse alveolar hemorrhage associ- ated with sevoflurane and cigarette smoking. J Community Hosp Intern Med Perspect 2019; 9(3): 247-51. https://doi. org/10.1080/20009666.2019.1608140*
3. *
  Lara AR, Schwarz MI. Diffuse alveolar hemorrhage. Chest 2010; 137(5): 1164-71. https://doi.org/10.1378/chest.08- 2084*
4. *
  Khanna AK, Cummings KC 3rd. Pulmonary hemorrhage in an outpatient ophthalmic anesthesia setting - it’s never ‘’just a cataract’’. J Anaesthesiol Clin Pharmacol 2012; 28(4): 520-3. https://doi.org/10.4103/0970-9185.101947*
5. *
  Kim CA, Liu R, Hsia DW. Diffuse alveolar hemorrhage induced by sevoflurane. Ann Am Thorac Soc 2014; 11(5): 853-5. https://doi.org/10.1513/AnnalsATS.201402-067LE*
6. *
  Mersh R, Ross C. Postoperative diffuse alveolar haemor- rhage: Insidious negative pressure or sevoflurane induced? BMJ Case Rep 2018; 2018: bcr2017222010. https://doi. org/10.1136/bcr-2017-222010*
7. *
  Cengiz O, Kivrak A, Yegen M, Demir M. Sevoflurane induced diffuse alveolar haemorrhage in a young patient after orthopedic surgery: A case report. Niger J Clin Pract 2020; 23(1): 120-2. https://doi.org/10.4103/njcp. njcp_51_19*
8. *
  Austin A, Modi A, Judson MA, Chopra A. Sevoflurane induced diffuse alveolar hemorrhage in a young patient. Respir Med Case Rep 2016; 20: 14-5. https://doi. org/10.1016/j.rmcr.2016.11.001*
9. *
  Ahmed-Khan M, Moin K, Funk C, Sachdev M, Jiffry MZM. Sevoflurane-induced diffuse alveolar hemorrhage. Arch Clin Cases 2023; 10(1): 29-31. https://doi. org/10.22551/2023.38.1001.10235*
10. *
  Holaday DA, Smith FR. Clinical characteristics and bio- transformation of sevoflurane in healthy human volunteers. Anesthesiology 1981; 54(2): 100-6. https://doi. org/10.1097/00000542-198102000-00002*
11. *
  Fatheree RS, Leighton BL. Acute respiratory distress syn- drome after an exothermic baralyme-sevoflurane reaction. Anesthesiology 2004; 101(2): 531-3. https://doi. org/10.1097/00000542-200408000-00034*
12. *
  Wu J, Previte JP, Adler E, Myers T, Ball J, Gunter JB. Spontaneous ignition, explosion, and fire with sevoflurane and barium hydroxide lime. Anesthesiology 2004; 101(2): 534-7. https://doi.org/10.1097/00000542-200408000- 00035*
13. *
  Kim JP, Park JJ, Kim NJ, Woo SH. A case of diffuse alveolar hemorrhage after tonsillectomy. A case report. Korean J Anesthesiol 2012; 63(2): 165-8. https://doi.org/10.4097/ kjae.2012.63.2.165*

